# Integration of Ubuntu philosophy in maternity care units of Limpopo province, South Africa: Perceived experiences of women

**DOI:** 10.4102/phcfm.v18i1.5240

**Published:** 2026-04-01

**Authors:** Seani A. Mulondo, Sonto M. Maputle

**Affiliations:** 1 Department of Advanced Nursing Science, Faculty of Health Sciences, University of Venda, Thohoyandou, South Africa

**Keywords:** integration, maternity, midwives, practice, Ubuntu philosophy, women

## Abstract

**Background:**

In South Africa, midwives should integrate the Ubuntu philosophy in maternity care services to promote utilisation of maternal and child healthcare services. Women expect to be treated with respect, love, dignity and mutual caring during childbirth. However, women experience obstetric violence, such as physical and verbal abuse during labour, which is associated with poor integration of the Ubuntu philosophy in maternity care units.

**Aim:**

The study aimed to explore and describe the perceived experiences of women during labour regarding the integration of Ubuntu in maternity services.

**Setting:**

The study was conducted in the six community healthcare centres that were purposively selected in Limpopo province.

**Methods:**

A qualitative, exploratory descriptive design was used. A non-probability, purposive and convenience sampling method was used to select participants. Data were generated from 24 women using individual face-to-face interviews. Trustworthiness and ethical standards were considered and adhered to. Data were analysed using thematic analysis.

**Results:**

The findings revealed three themes associated with a lack of integration of Ubuntu when rendering maternity care services, and two sub-themes emerged from themes one and two; three sub-themes emerged from sub-theme three. First theme: the personal conduct of midwives as perceived by women during labour. Attitude of midwives towards women in labour and midwives’ lack of commitment emerged as sub-themes. Second theme: interpersonal relations as perceived by women during labour. Poor midwife-woman relationship and communication, and abusive conduct by midwives emerged as sub-themes. Third theme: the support system needed by women during labour. Poor support from midwives, the use of traditional remedies versus Western medicine, and a lack of sufficient resources that deprive women of quality midwifery care services emerged as sub-themes. Verbal and physical abuse, poor midwife-women relationships, use of traditional remedies and lack of support could lead to inadequate utilisation of maternity care services by pregnant women for childbirth.

**Conclusion:**

Integrating Ubuntu principles into maternity care can improve maternal and neonatal outcomes by promoting compassionate, respectful, and patient-centered care.

**Contribution:**

Reviewing the midwifery curriculum to include Ubuntu values will help student midwives apply these principles in clinical practice and foster a culture of respect and ethical care.

## Introduction

Globally, Ubuntu philosophy is the current theme for social work and social development, representing the highest level of global messaging.^1^ However, it originated in the African countries to shape the lives of people in the community to promote health. In maternity care units, the application of the African philosophy of Ubuntu improves maternal and child healthcare services (MCHCs).^2^ Ubuntu philosophy is comprised of values, beliefs, love, respect, wisdom, confidentiality and practices that enable midwives and women to be authentic human beings through interaction with each other in the maternity care units.^3^ Behaviours such as verbal and physical abuse, non-consensual care, non-confidential care, neglect, abandonment of care and bribery were reported as abuse of women in maternity settings by midwives worldwide.^4^ A lack of professional support for midwives, excessive workload, inadequate staffing and poor infrastructure are regarded as the main aggravating factors for women’s abuse.^5^ Similarly, the World Health Organization (WHO)^5^ also indicates that the abuse of women in maternity settings, especially during childbirth, is noticed through disrespect and abuse. That strongly supports the global phenomenon of a lack of Ubuntu in maternity care service facilities that need urgent attention.^6^

In midwifery practice, Ubuntu emphasises the application of principles of care, such as respect, kindness, dignity and compassion, to ensure the well-being of both mother and baby. Hence, the Ubuntu philosophy, women-centred approach and midwife-woman relationship are integrated into all aspects of midwifery practice. Midwives should uphold the dignity of individual pregnant women by showing respectful care, support, empowerment and being culturally sensitive to promote culturally competent midwifery care.^7^ In maternity care units, midwives must have a personal and professional philosophy of life to provide quality midwifery care during pregnancy and childbirth. The focus is not only on the well-being of mother and baby but also on promoting and improving the well-being of families, societies, communities and the environment. Midwives must view the meaning of their lives, attitudes and behaviours, as well as the lives of the women they serve, in relation to midwifery practice. Therefore, the practice of Ubuntu philosophy in maternity services is crucial to the nation’s well-being, as MCHCs are the priority, and urgent action should be taken.^8^

A study conducted in Abuja, Nigeria, reports that mistreatment of women during childbirth in maternity settings was evidenced by signs of violation of privacy, neglect and verbal and physical abuse. The report further concluded that some women were exposed to detainment because of failure to pay hospital fees.^9^ Diamond-Smith et al.^10^ report from the study conducted in the north-eastern state of India that the majority of women were punished by being hit with a stick because they soiled the bed linen with faeces, and the cutting of an episiotomy without giving anaesthesia. Another study conducted in Uttar Pradesh in northern India reported lack of privacy and confidentiality, physical violence, no consent for vaginal examination and shaving of the perineum in public hospitals and maternity wards.^11^

The different tribes in Southern, Central, Western and Eastern Africa incorporated Ubuntu into their daily lives and activities as a symbol of caring for others and in maternity care units.^12^ In Ethiopia and other low- and middle-income countries, studies conducted reported that disrespect and abuse of pregnant women when providing MCHCs services retard adequate utilisation of maternity care services.^13,14^

According to Davis-Floyd^15,16^ the lives of both mother and baby are in the hands of midwives from conception until delivery of the baby. The birth of a child is more than life itself. Midwives must always consider the key principles of nursing ethics, such as non-maleficence, beneficence, autonomy and justice, which are regarded as fundamental when rendering maternity care services in public healthcare facilities.^17^ The South African Nursing Council (SANC) in South Africa also recognises and recommends that all nurses apply these principles while providing nursing care to people; hence pregnant women are not left outside. The application of Ubuntu philosophy in maternity care services is evidenced by the principles of caring, such as respect, dignity and compassion, and emphasises caring as the core in the midwifery practice.

In South Africa, maternity care services are rendered freely by competent midwives at primary health care (PHC) facilities, district hospitals and tertiary hospitals for the best maternal and neonatal health outcomes.^18^ Studies conducted focused on the experiences of midwives in providing care for labouring women, barriers and facilitators influencing midwives in the implementation of maternity care services and experiences of male student nurses in maternity care.^5,19,20^ Insufficient studies might have been conducted on respectful care and the practice of a women-centred approach in maternity care services. This study could add value to the existing knowledge base by integrating the Ubuntu philosophy into maternity units, as practised by midwives. The integration of Ubuntu philosophy in maternity care units should focus on providing high-quality maternity care, which is based on principles of fairness, responsibility, accountability, love, respect, helpfulness, compassion, sharing, trust and integrity. Midwives should integrate Ubuntu principles into maternity care services to promote the adequate utilisation of antenatal care (ANC), labour and postpartum care. Pregnant women expect to be treated with respect, love, dignity and mutual caring. In the midwifery context, Ubuntu must be seen as the act of being human and midwives expressing compassion, caring, dignity, sympathy, harmony, empathy and forgiveness towards pregnant and breastfeeding women.^21^ However, women experience obstetric violence, such as physical and verbal abuse during labour and childbirth, which is associated with poor integration of Ubuntu philosophy in maternity care services. The aim of the study is to explore and describe the perceived experiences of women towards the integration of Ubuntu philosophy in maternity care services by midwives.

## Research methods and design

### Study design

A qualitative, exploratory descriptive design was used to obtain in-depth information from the participants. This produced the thick description of participants’ feelings and experiences, and the meanings of their actions were interpreted.^22^ The researchers explored and gained more information on the perceived experiences of women regarding the integration of Ubuntu philosophy in maternity care services to have a better understanding of the existing problem.

### Setting

The study was conducted in the six community healthcare centres (CHCs) that were purposively selected from Limpopo province, providing maternity care services. Limpopo province comprises 5 districts, 44 hospitals, 145 clinics and 10 CHCs. Community healthcare centres provide MCHCs, geriatric services, psychiatric services and management of minor ailments. Each district has two CHCs providing maternity care services. Because of limited resources, such as manpower, cost and time, researchers were unable to conduct the study throughout the entire province of Limpopo. Six CHCs were selected from three districts, namely Vhembe, Mopani and Sekhukhune, to cover the three most spoken languages in Limpopo province, that is, Tshivenda, Sebedi and Xitsonga. The selection was based on the high statistics of women who have delivered in those CHCs. Legal cases associated with mistreatment, physical and verbal abuse and poor communication between midwives and women were reported from the three selected districts in Limpopo province.^23^

### Population and sampling strategy

The population comprised all women who had given birth to babies at selected CHCs. The selection of participants was based on the information required by the researchers to answer the research question and gain in-depth insight into achieving the study objectives. The researchers gained a deep understanding of the complex matter regarding women’s experiences on the study topic, specifically the integration of Ubuntu in maternity care units, using a small sample size. The target population was women who gave birth at the selected CHCs. The accessible population was those women who agreed to participate in the study. Non-probability, purposive and convenience sampling methods were employed to select 24 participants who were present during the data collection process. Recruitment was conducted through the operational manager of the maternity section, and women were personally contacted by the researchers. Women who have given birth, aged 20 years and above, were included in the study and regarded as having experience with pregnancy, labour and childbirth. Primigravida women who had not yet given birth were excluded from the study. Adherence to the inclusion criteria would protect the participants, as some studies might be harmful to them, especially those involving underage participants. Participants could also be mature enough to be able to respond appropriately to the research questions.

### Data collection process

Two female researchers were involved in data collection. One of the researchers was a registered specialised midwife in South Africa, a Doctor of Philosophy (PhD) holder with extensive clinical and academic midwifery experience; the other one was an academic with a PhD and had significant academic and clinical midwifery experience. Data were generated from 24 women using individual face-to-face interviews. The data collection process was conducted from 01 May 2023 to 30 May 2023 in six selected CHCs from the three districts of Limpopo province. An unstructured interview was conducted. One central question was asked as follows: ‘Share with me your perceptions and experiences on integration of Ubuntu in maternity care services by midwives’, and it was followed by probing to obtain a deeper understanding of the experiences of women during maternity care services. Pre-testing was conducted with three participants from one CHC, which did not form part of the study, to determine whether the question was clear, relevant and effective in eliciting the desired information from the target population. The findings were added when analysing the data. The researchers explained the purpose of the study, its benefits and the counselling to be provided in case of emotional stress during the interview, as well as the procedures for the interview. Translation and back translation of the question were performed into three common spoken languages in Limpopo province, Tshivenda, Sepedi and Xitsonga to accommodate women who could not understand or speak English. Participants were asked to voluntarily sign the consent forms before the commencement of the interviews. The interviews were recorded using an electronic voice recorder, and a verbal agreement for its use was requested by the participants. All participants agreed to the use of the voice recorder; none declined, instead. This ensured that the data recorded were captured accurately. Detailed field notes of the conversation were taken by the data collector during the interview session and consolidated immediately at the end of the day to avoid forgetting some critical aspects of the data. An interview was conducted for a period of 30 min – 45 min with each participant in a quiet room, free from external disturbances. Data saturation was employed because it focuses on assessing the sample size rather than the adequacy of the data to develop a theory.^24^ Data were collected until data saturation was reached at participant 20, but researchers continued with an additional four participants. No new insights were identified, and repetition of data indicated that an adequate sample size had been reached. To ensure the validity of the study’s findings, member checking was carried out. Those selected to participate in the study, none refused, and no repeat interviews were conducted.

### Data analysis

The data were analysed using inductive thematic analysis. Analysis was performed simultaneously with data collection. The second author led the data analysis, as the researcher is an expert in the qualitative approach, aided by input from the first author. The data derived from the interviews were transcribed verbatim and translated from Tshivenda, Sebedi and Xitsonga to English to detect other elements, including non-verbal cues, pauses and laughing, as well as to confirm whether these signs were present in the transcripts. The recorded interviews were listened to in tandem with the field notes. The translated interviews were analysed using the inductive thematic analysis technique. This led to themes and codes that emerged directly from the data without preconceived ideas, as described in Creswell and Creswell,^25^ by reading each transcript separately, and a general understanding of the material was gained. The transcripts were then repeated, with important sections highlighted with various colours. Based on the colour coding, the underlined statements were grouped and assigned a theme. Related themes were combined, and the procedure was updated. After reviewing the transcripts, a separate coder created themes and sub-themes. After comparing and discussing the parallels and differences, a consensus was achieved.

### Measures to ensure trustworthiness

Criteria such as dependability, credibility, transferability and confirmability were attained to achieve trustworthiness as described by Lincoln and Guba.^26^ Credibility was established through a trusting relationship with the participants, enabling them to talk freely, and through prolonged engagement by spending an extended period of time during data collection. Participants were allowed to share their perceptions of the phenomenon under study to help the researchers gain an in-depth understanding. Member checking was done to confirm the data collected from participants. Triangulation was achieved through multiple methods of data collection, including in-depth individual interviews, observations, and field notes, to ensure the validity and reliability of this qualitative research study. Dependability was ensured through checking the findings with the participants and keeping an audit trail for use, the coding and re-coding procedure, and an independent coder was used to analyse the data. Conformability was ensured by providing evidence that supports the findings and interpretations by means of auditing and triangulation for the accuracy of data collected. Lastly, transferability was observed by ensuring that the results of the study were accurately collected, so that they could be applied to similar situations that might occur in the future.

### Ethical considerations

Ethical clearance to conduct this study was obtained from the University of Venda Research Ethics Committee (No. SHS/20/PDC/34/0511) and the Department of Health in Limpopo province granted permission to conduct the study. This study was conducted according to the Declaration of Helsinki. Ethical standards, including informed consent, anonymity, confidentiality and voluntary participation, were adhered to. Written informed consent was obtained from all participants who volunteered to participate in the study prior to their involvement. Participants have the freedom to leave the study at any moment, free from coercion or undue influence.^27^

## Results

### Demographic characteristics of participants

Twenty-four women participated in the study. The participants ranged in age from 21 years to 41 years and above. Fourteen participants fell within the 21–30-year age bracket, 6 were in the 31–40-year age bracket and 4 were 41 years old and above. The majority of the participants, 19, were living without a spouse, and only 5 were married. Regarding the area of residence, 18 came from rural areas, and only 6 were from urban areas.

### Presentation of findings

Three key themes emerged, namely: (1) personal conduct of midwives as perceived by women, (2) interpersonal relations as perceived by women and (3) support system needed by women. Two sub-themes emerged from the first two themes, and three sub-themes emerged from the third theme. The themes and sub-themes that emerged from the perceived experiences of women regarding the integration of Ubuntu by midwives in maternity units are presented in Table 1.

**TABLE 1:** Themes and sub-themes: Perceived experiences by women on integration of Ubuntu by midwives in maternity units.

Themes	Sub-themes
1. Personal conduct of midwives as perceived by women during labour	1.1 Attitude of midwives towards women in labour. 1.2 Midwives’ lack of commitment.
2. Interpersonal relations as perceived by women during labour	2.1 Poor midwife-women relationship and communication. 2.1 Abusive conduct by midwives.
3. Support system needed by women during labour	3.1 Poor support from midwives. 3.2 Use of traditional remedies versus Western medicine. 3.3 Lack of sufficient resources depriving quality midwifery care services.

### Theme 1: Personal conduct of midwives as perceived by women during labour

Most women expressed various factors associated with the personal conduct of midwives that affect the integration of the Ubuntu philosophy in maternity care services. Attitude of midwives and lack of commitment emerged as sub-themes.

#### Sub-theme 1.1: Attitude of midwives towards women in labour

Different attitudes of midwives as perceived by women were expressed as affecting the integration of Ubuntu at ANCs and during labour, such as a lack of professional secrecy, ignorance, rudeness and harshness. One participant who has experience in pregnancy and childbirth expressed the mistreatment from the midwives she received when reporting labour pain in the maternity unit:

‘You know, as a woman who has experience with childbirth, I do not rush to the maternity care unit on the commencement of labour. Pause, on my arrival at the labour ward, I was given a bench and sat there for almost two hours without being checked! Nurses were coming in and out and not bothering to assist or talk to me!’ (P4, female)

Another participant expressed how she was dehumanised by midwives as she was called by the number of children or pregnancy, and that made her feel embarrassed in front of other women:

‘I am reluctant to report early for labour pains. Instead of calling us names, nurses usually say that one has a twin pregnancy or five children. I was coming to give birth to my fifth child, and one nurse asked me loudly: Are you coming to deliver your fifth child? Do you need to have a span for children? and I was so embarrassed!’ (P14, female)

Another participant expressed similar feelings regarding the negative attitude of midwives in the maternity unit. The participant indicated that midwives are rude and do not provide privacy:

‘We are experiencing a different negative attitude from midwives; their tone and language when talking to us is very bad! Pause, there is no privacy at government clinics and hospitals! Rude, especially in the maternity labour unit! It is so humiliating!! There is no Ubuntu! I tell you because of what I discovered during my child’s birth. I called for an assistant when the water broke out and was dripping down, and I was ignored! No one came to me.’ (P23, female)

The findings could affirm that most of the midwives have a negative attitude towards labouring women. This could be associated with the non-practice of a women-centred approach, where the comfort, autonomy and preferences of women are not taken into consideration. This shows that midwives have no professional secrecy and an explanation of the labour processes as a sign of Ubuntu when rendering maternity care services.

#### Sub-theme 1.2: Midwives’ lack of commitment

Most women expressed that midwives do not have a caring concern, which was supported by the following quotes.

One participant expressed her experience during labour by indicating that midwives do not care about labouring women. Midwives ignore or do not respond to a request from labouring pregnant women. Midwives neglect, and the women end up giving birth on the floor, or they may come only when one is screaming about the crowning of the baby’s head:

‘Labour pains are too painful, but they are worse when you are being assisted by midwives who don’t care! Those midwives who work during the night do not care. They will only come to you when you scream that the baby’s head is coming out. I remember when a pregnant woman gave birth on the floor! It was so humiliating to the delivered woman, what worried mostly was that midwives put the blame on the woman! There was nothing the poor woman could do if not provided with a bed.’ (P21, female)

Another participant expressed non-assistance by midwives when labouring women are requesting painkillers in case of severe, strong labour pains or requesting for operation. Midwives usually tell women that the baby will come out through its entrance to the womb:

‘I asked for a painkiller because I couldn’t stand those severe labour pains. I also requested the operation because I spent the whole day in the labour room, in pain! Instead of being given the medication, I was told that the how the baby got through to me will come out that way!’ (P6, female)

Another participant expressed that midwives do not provide pregnant and labouring women with information about pregnancy and childbirth, including what to expect and how they are expected to behave. Monitoring during labour is not ideal because women can only be checked once:

‘Hmmm! Since I arrived at the labour unit, I was checked only once! When the doctor came to see the other women, he was told that there was no need to see me! I was neglected and abandoned because they said that I was not cooperating! I was never given any information regarding my labour process! Because of what I went through, I may not like any of my relatives to come for delivery in this facility!’ (P8, female)

The findings revealed a lack of commitment as perceived by pregnant women that could compromise the quality of midwifery care during labour, leading to poor maternal and neonatal outcomes. Pregnant women expressed feeling left unattended, disrespected and their requests ignored by midwives. Midwives who are not committed to their midwifery practice cannot practice Ubuntu in their daily midwifery practice. Instead, they display their uncaring behaviour that destroys the morale of pregnant women during labour.

### Theme 2: Interpersonal relations as perceived by women during labour

Ubuntu philosophy emphasises the relationship of mutual respect, compassion and dignity between pregnant women and midwives, leading to positive maternal and neonatal outcomes. The following sub-themes emerged.

#### Sub-theme 2.1: Poor midwife-women relationship and communication

Poor relationships between women and midwives play a role in the underutilisation of ANCs and delay in seeking obstetric care during labour, as expressed by most women in labour. Most participants alluded to the challenges of transportation, which often cause women to arrive late for ANC services. Although some midwives are friendly, others are reportedly unfriendly. Midwives often struggle to listen to women, which can result in poor communication.

‘Iyaaa!!, Yo! Some midwives are not friendly at all! They do not want to listen to us if we arrive late at the clinic! Some of us are from deep rural areas where there is no transport! Pause, there is only one lane for buses which leave the village very early, around 05h30! And once it has gone, it is over, and there is no other means of transport!’ (P10, female)

Another participant further expressed that they are not cared for and respected as pregnant women because midwives do not want to listen to their concerns. If one breaks the clinic rules, such as arriving late, they are sent back and must come the following day without assistance. This is a concern as pregnant women are mistreated even during their pregnancy:

‘Failure to comply with the clinic rules, you do not get ANC service, and you are sent back home to come back the following day. Really, communication is very poor! No one listens to you, and that makes pregnant women not share their pregnancy-related problems. Pause, this is also a sign of disrespect to us as women!!’ (P1, female)

Another participant shared her experience during the coronavirus disease 2019 (COVID-19) pandemic, indicating that midwives did not have sufficient time to conduct thorough examinations. In 5 min, they would be finished with pregnant women during pregnancy and labour. Some midwives could have enough time with a pregnant woman. Based on that, pregnant women mostly enquire about the midwives on duty before seeking pregnancy-related care:

‘Nurses never have time to sit and explain clinic activities with us! Pause, this was worsened by the COVID-19 disease! In five to ten minutes, they are done with the abdominal check. Shooo!! Because of poor relationships among some of the nurses, especially those who assist us during birth, the majority of us usually enquire about the midwife who is on duty because some are kind and approachable, and we seek maternity care services during their presence!’ (P16, female)

Another participant expressed that poor relationships between pregnant women and midwives prevent pregnant women from disclosing some of their pregnancy-related problems, such as the mode of previous childbirth. Pregnant women need to be respected and trusted:

‘That lack of mutual relationship of trust, respect and dignity makes us as pregnant women not open and disclose some sensitive information with midwives, for example, if I have delivered two children by operation, I may not disclose to midwives, and that in turn could lead to mismanagement and lead to losing a baby, or something bad can happen to me.’ (P20, female)

The findings of this study revealed the poor women-midwife relationship, such as unfriendliness, disrespect, mistrust and not being listened to by midwives. The poor midwife relationship disadvantages pregnant women from receiving health education regarding labour processes; hence, women are reluctant to share their feelings with midwives, leading to emotional stress that could cause postpartum depression and poor neonatal bonding. The findings further indicated that women might prefer to visit other clinics where there is a friendly, mutually respectful and dignified relationship with the midwife on duty.

#### Sub-theme 2.2: Abusive conduct by midwives

Participants expressed that they were exposed to verbal and physical abuse during their childbirth in maternity units.

**Physical abuse:** Most participants expressed that in some of the maternity obstetric units, women experienced neglect, physical abuse, abandonment during labour and improper naming. This is supported by the following quotes from the participants.

One participant, who was a multiparous woman, expressed her painful experience during childbirth. The participant expressed physical abuse through the clapping of her labia minora with an instrument. The participant further alluded to seeking another health facility for her next pregnancy and could discourage other pregnant women from visiting the health centre concerned:

‘I have experienced a painful delivery during my fifth pregnancy! Looking down and further said: I may not forgive that midwife! You know, my thighs were slapped and clapped my labia with a certain steel that I didn’t notice what that was!!, Yooo!! It was so painful! Pause, my challenge is that I am from one of the poorest families. I could continue with pregnancy-related issues through private or special doctors; however, financially, I cannot afford it! We depend on child grants for survival as my husband is not working!’ (P7, female)

Another participant expressed the discomfort experienced during labour. Participant indicated that midwives were very vigorous when inserting their fingers through the vagina to check the opening of the child’s way for birthing. That was not a pleasant experience:

‘There is no tender care or compassion when midwives are checking how the baby is lying inside the abdomen and during examination through the vagina! The way they touch your abdomen is not at all comfortable! And even when they insert their fingers, it’s not comfortable!’ (P17, female)

Another participant expressed a similar opinion that midwives are not compassionate when treating pregnant women during labour. This is the reason why some pregnant women delay visiting the maternity unit during labour. Those who are experienced with childbearing usually report a head-on perineum:

‘Most nurses do not have compassion, but I don’t have a choice because I need to be assisted with childbirth! It is so discouraging to visit the labour unit earlier; instead, I have to wait at home and only come when I can feel that the baby is ready to be born!’ (P19, female)

During pregnancy and when in labour, midwives do not explain the instruments they use for foetal monitoring. The participant indicated that the instrument is uncomfortable when placed on top of the abdomen. But some midwives do explain the procedures before starting, and this makes us at ease:

‘Midwives just put a mill-like structure on top of the abdomen, not telling you what she is doing, kneading our abdomen vigorously! On top of all these, there is no explanation given to you as a woman coming for the birth processes! However, some do explain each step!’ (P3, female)

**Verbal abuse:** Most women, both nulliparous and multiparous, experienced much of the verbal abuse. One participant expressed that midwives are rude, can punish and say vulgar words, in a high tone to labouring women, despite the availability of non-nursing staff like cleaners. That mostly humiliates pregnant women during childbirth. However, pregnant women do not have any means of defending themselves:

‘Midwives are rude; they scold us, rebuke, or punish us. I remember after giving birth, the cleaners were not around, and I was instructed to carry my dirty, bloodstained sheets out of the bed!’ (P24, female)

Another participant further indicated that pregnant women are reluctant to report labour pain in advance because they are afraid of being hurt by words during labour:

‘You know, pregnant women delay in reporting labour pains because they are afraid to be scolded with that harsh tone! It is very embarrassing because it is done in front of other labouring women!’ (P11, female)

Participants reported more mistreatment, disrespectful care, non-attendance during labour and a lack of communication:

‘Midwives are rude! They lack a caring heart! Midwives delay in attending to new arrivals in the labour ward, despite the strong labour pains. I was given a bench, and I sat on it! And when I requested assistance, The midwife told me, “Can’t you see that I am still busy with another woman who arrived earlier than you?” Our right to proper midwifery care is not considered.’ (P5, female)

The pregnant women expressed that labouring women are verbally and physically abused by midwives during childbirth. Midwives are rude, harsh, scold, impatient, shout, disregard the cultural and religious beliefs of women and judgemental to women with higher parity or multiple children of five and above. Midwives should apply the principles of Ubuntu in maternity care, treating labouring women with kindness, empathy, compassion and respect. Pregnant women also lack knowledge of pregnancy-related management and childbirth.

### Theme 3: Support system needed by women during labour

Women in labour need to be supported emotionally, psychologically and empowered with knowledge related to pregnancy and childbearing by midwives to promote their spiritual well-being. The following sub-themes emerged.

#### Sub-theme 3.1: Poor support from midwives

Women expressed that support from midwives and even from other health professionals is poor. There is no practice of Ubuntu philosophy in maternity care services. Failure to get support could lead to postpartum depression. This is supported by the following quotes from participants who expressed a lack of support from midwives during labour, as multiparous women are discouraged and tend to report late during pregnancy and labour:

‘What I have noticed is that, as this is not my first pregnancy, most midwives may give fake support, which discourages women from presenting early for childbirth. Others, especially those with five children or more, may not book for pregnancy care, not attend ANCs, and come to the labour unit towards delivery.’ (P13, female)

Another participant further expressed that poor support from midwives leads to a reluctance to visit the ANC clinic regularly. Instead, pregnant women could only come for the maternity case record book and return when they were in labour. It is further alluded that midwives were those who used to visit women to their homes after birth and gave care to both mother and baby:

‘The majority come for a clinic visit only once to get that book for going to the clinic or hospital when in pain, for giving birth, or because of a lack of support from midwives. Participant further said that: midwives are those from olden days who used to come to houses where there was a newborn, carrying that bag to check and assist the woman, not these days!!!’ (P15, female)

Other participants had concerns about social media, as it is the one that disturbs midwives from providing respectful maternity care, characterised by kindness, love and comfort:

‘Haaa!! These days! These nurses do not have time to assist us! Maybe in other maternity care facilities, I do not know! Where will the cell phones be? Yooo!! They are always busy sharing videos. You are in labour; no one will come to you! Instead. You will be told to shut up because you are making noise! You must wait for those 2–4 hours of re-checking! Clamped her hands!’ (P22, female)

The findings revealed that poor support from the midwives pushes pregnant women to visit the ANCs only once for the purpose of getting the maternity case record. Social media has taken the lead, and the majority of middle-aged women keep themselves busy on social media and neglect labouring women. Labouring women lose trust and seek maternity care services from another maternity care facility.

#### Sub-theme 3.2: Use of traditional remedies versus Western medicine

Women have their own rights, such as cultural rights and the right to make their own choices. Therefore, midwives need to integrate these into the Ubuntu when providing maternity care services without any discrimination. Women expressed that there are women who turn to traditional remedies and abandon Western medicine or accessing PH facilities or hospitals for pregnancy and labour matters because of discrimination. One of the multiparous women had a concern about traditional remedies, such as Isihlambezo, that stimulate and facilitate labour. Pregnant women need their cultural beliefs to be considered for pregnancy and childbirth:

‘You know, midwives delay us in delivering the baby because they deny us the right to push the baby, pause, they do not want to consider our cultural beliefs regarding pregnancy and childbearing.’ (P2, female)

Participant further said that:

‘[*O*]nce at term, we drink traditional medicines/remedies [*Makgorometja, Isihlambezo or boiled water from Ostrich eggs*] to stimulate and facilitate labour.’ (P2, female)

The findings revealed that multiparous women are the ones who use traditional remedies for pregnancy and labour because they have experience with pregnancy. Multiparous pregnant women do not seek maternity care services early, but they progress themselves and present to the labour unit when ready to give birth. Poor accommodation of their cultural beliefs and the use of traditional remedies for labour induction led to mistreatment during childbirth.

#### Sub-theme 3.3: Lack of sufficient resources depriving quality midwifery care services

Women expressed concerns about the unavailability of essential resources, such as delivery beds, sanitary towels, baby kits and a Baumanometer. One participant was unpleasant about the situation experienced, where there were no delivery beds in the health centre. That made pregnant women in labour be monitored, and their labour progressed while roaming around and sitting on the bench:

‘The unavailability of delivery beds was a challenge; midwives don’t tell us that beds are only for those who are giving birth! Only two beds are available in this maternity care unit, and eight to ten women give birth each day. Therefore, we had to sit on a hard bench until it was time to give birth to a child! Some gave birth on the floor.’ (P9, female)

Another participant further expressed that sometimes they were told to bring their own sanitary towels and disposable nappies for the newborn because of the insufficiency in the maternity unit. That is a concern because those who cannot afford it are at risk:

‘Midwives do not tell us in time regarding the shortage of sanitary towels and baby kits/attire, you are given only one sanitary towel and when you ask for the second one, is then that you are told to send someone buy on your behalf. As you know that some of us are poor, we cannot afford to buy, hence a huge challenge!’ (P12, female)

Participants expressed concern that the unavailability of delivery beds is a significant issue, as they are only placed on a bed during examination. Not all pregnant women can afford to bring their own sponge to the maternity unit:

‘The very most frustrating and degrading us, is when you are in labour, you are only allowed to climb the bed during checking!! Shooo! Some pregnant women end up delivering on the floor, and it’s so humiliating! One bed is used by multiple pregnant women in labour! At least if they allow our relatives to bring a sponge to lie on it for one’s comfort!’ (P18, female)

The findings revealed a shortage of human and material resources in maternity care units. Midwives often fail to explain the unavailability of essential resources, such as sanitary towels and disposable baby nappies, as well as the shortage of staff.

## Summary of the findings

The findings of the study revealed that inadequate application of the integration of Ubuntu in maternity care units is associated with the personal conduct such as the negative attitude of midwives towards women and their lack of commitment in providing maternity care services; interpersonal relations as perceived by women during labour such as poor midwife-women relationship and communication and abusive conduct by midwives; support system needed by women during labour such as inadequate support from midwives, use of traditional remedies versus Western medicine and lack of sufficient resources depriving quality midwifery care services.

## Discussion

Maternity care services are not fully incorporating the Ubuntu philosophy, as affirmed by the majority of women. Women were experiencing a negative attitude from midwives, verbal and physical abuse, a lack of support and a poor relationship between women and midwives. That could make women feel embarrassed, ashamed and humiliated among women coming for childbirth in maternity units. That could further discourage women from reporting commencement of labour pains, women could progress their labour in their respective homes and report when they experience rupture of membranes.^28,29^ Similarly, Malatji and Madiba^4^ report that unacceptable conduct by midwives includes shouting at a distance when talking to women, discrimination and being labelled by the number of children when called. This could indicate that women are disrespected and mistreated during the process of labour and childbirth. This is unacceptable according to the ethical code of conduct for midwives in South Africa.^30^

Despite the guidelines for maternity care in South Africa, which stipulate that midwives should treat women in labour with respect, privacy and courtesy as the basis of Ubuntu philosophy, women experience disrespect and abuse by midwives.^8^ Ignorance and abuse are the major contributors to disrespectful maternity care during childbirth. Similarly, National Integrated Maternal and Perinatal Care Guidelines,^31^ indicated that violation of human rights among pregnant women during childbirth is associated with disrespectful care, abuse and other mistreatment, leading to mental stress to labouring women. Women could be discouraged from seeking maternity care services for childbirth earlier. Hence, it could lead to maternal and neonatal complications such as pre-eclampsia and low Apgar score and neonatal asphyxia, as the women would not be managed appropriately during the first stage of labour and childbirth.^8^ According to the National Department of Health, MCHCs are regarded as a priority area requiring urgent attention in South Africa.^8^ The neglect and abandonment were revealed as common problems experienced by most pregnant women. Most midwives no longer possess the caring and compassionate spirit of Ubuntu when providing maternity care services, despite litigation and lawsuits resulting from the improper management of maternity cases in labour units. Women gave birth alone without the assistance of midwives, and some sustained third-degree tears.^4^ Another study revealed that neglect was mostly experienced by young women aged 18–25 years. Midwives from that community were used for caring for women who were in the age brackets of 25–40 years.^32^ The study conducted in Ethiopia reports that women who gave birth unattended sustained second- and third-degree tears. Some of those women refused to be sutured because they were afraid of severe pain, as they might not be given painkillers or local anaesthesia before suturing.^33^ Therefore, the practice of Ubuntu philosophy in maternity care units is a global concern although it originated in African countries. In another study, it is reported that midwives failed to integrate Ubuntu into maternity care units during labour and childbirth because of high numbers of labouring women; midwives could not monitor the progress of labour regularly, and some gave birth unattended. This led to increased maternal and neonatal mortality, increased medico-legal hazards in the maternity care unit, with compromised midwifery practice.^5^

Contrary to this study’s findings, Atmuri et al.^34^ report a positive attitude among midwives, as manifested by sympathy, love and care during labour. During labour, women were reassured that the best would be provided to ensure that a healthy and normal baby would be delivered. The partner was invited to be next to the labouring woman, and any poor progress was reported to the obstetrician; hence, the midwife acts as an advocate for the labouring woman. The practice of a women-centred approach in maternity care units allows women to make their own choices or decisions regarding their mode of delivery. Midwives should empower pregnant women with pregnancy-related information, such as potential procedures and childbirth. Women should be treated with sympathy and non-judgemental care.

A unique, good woman-midwife relationship is a significant factor in providing effective maternity care services to improve the social, physical, psychological and emotional well-being of women.^35^ Midwives should present the care, compassion, communication, commitment and competence as an integration of Ubuntu philosophy in maternity care services to promote the well-being of both mother and baby. Thus, it reduces the maternal and neonatal mortality.^5^ The study conducted in Western Australia revealed that women-centred care was found to be the essential construct for midwives when providing maternity care services to women. However, the identified poor relationship of undignified and disrespectful treatment during labour was addressed to promote quality midwifery practice.^36^ In this study, the outbreak of COVID-19 could have aggravated the poor women-midwife relationship and communication. That could have further distorted the integration of Ubuntu because of a poor relationship. It was noted that caring for a pregnant woman with COVID-19 by midwives was a huge concern as it could be associated with fear of casual contact. The study conducted in Melbourne, Australia, revealed consistent findings that midwives were not comfortable in taking care of women who contracted the COVID-19 pandemic.^37^

The physical and verbal abuse of women during labour and childbirth could be classified as obstetric violence that occurs in maternity obstetric units or PHC facilities where deliveries are conducted. That is a concern because pregnant women are vulnerable to physical and psychological trauma associated with the non-practice of Ubuntu in midwifery care. Nolte and Downing^38,39^ reported that the lack of Ubuntu in maternity services was manifested by dehumanisation and disrespectful care during pregnancy and labour. Denial of women to make informed decisions about their bodies and mode of delivery has also affected their mental state. Women who are disrespected and abused in all forms could be discouraged from seeking maternity care services, leading to late presentation at the labour unit for childbirth. Consequently, women could choose to undergo an elective caesarean section to avoid humiliation and postpartum depression, which could also be denied by midwives, as expressed by participants in this study. Women need to be given support during pregnancy, labour and the postpartum period.

Women at advanced maternal age (40 years and above) and grand multiparous mothers could tend to seek traditional birth attendants for traditional remedies to induce labour to overcome such kinds of abuses by midwives. In Ethiopia, it was reported that pregnant women from remote rural communities use herbal remedies for the induction of labour, to reduce labour pains and to increase the desire to push during childbirth. However, those remedies could precipitate labour that could lead to antepartum haemorrhage and rupture of the uterus, leading to maternal or neonatal death. The traditional remedies were not scientifically tested and integrated by health professionals.^39^ Women seemed to be more influenced by local practices than by modern scientific medicine because of a lack of Ubuntu in maternity care settings. In Limpopo province, pregnant women visit TBAs who give them traditional herbs called Isihlambezo and Ostrich egg to stimulate and facilitate labour.^40^ It is found that in some areas, traditional birth attendants (TBAs) have been recruited to perform tasks related to Prevention of Mother-To-Child Transmission (PMTCT), discouraged from the use of herbal remedies to stimulate labour and trained to provide pre- and post-test counselling and refer pregnant women to maternity care units for management of labour.^41^

Health providers, especially midwives, are the core providers of MCHCs worldwide.^42^ Midwives are responsible for portraying a woman-centred approach and creating an environment conducive to a mutual relationship of trust and respect. This would encourage and motivate mothers to feel at ease, comfortable and open to midwives, as the majority of babies are delivered by them.

### Strengths

The integration of Ubuntu philosophy into maternity care services could promote adherence to and implementation of ethical principles, such as respect, confidentiality, courtesy, compassion and the midwife-woman relationship. This would improve the delivery of quality MCHCs in PHC facilities, district and tertiary hospitals of Limpopo province, South Africa. Hence, improve the adequate utilisation of maternity care services that would promote the well-being of both mother and baby, as the users of these services. Midwives could be empowered during on-the-job training on the integration of Ubuntu philosophy in maternity care services to reduce unethical behaviour of verbal and physical abuse that are most common in maternity units.

### Limitations

The sample size of 24 participants is too small for the Limpopo province, which comprises five districts. However, this was informed by data saturation. The study was conducted in only six CHCs; hospitals were not included. Therefore, the findings of this study cannot be generalised to other districts or provinces in South Africa.

### Recommendations

There is a need to conduct further research on strategies to improve the integration of Ubuntu philosophy and adherence to ethical issues in maternity care services, experiences of midwives during management of pregnant women in labour units for childbirth in Limpopo province, South Africa. A review of the midwifery curriculum and integration of the values of Ubuntu in its principles, purpose and aims in the midwifery curriculum for student midwives to apply knowledge and skills during midwifery clinical learning. The re-establishment of community mobile maternity care services by community healthcare centres to improve the health status of both mother and child, thereby reducing the maternal and neonatal mortality.

## Conclusion

Disrespect, poor midwife-women relationship, verbal and physical abuse of women in maternity healthcare units are the most common factors associated with a lack of Ubuntu in maternity care services and need urgent attention to address. Midwives are encouraged to integrate and practice the philosophy and values of Ubuntu in maternity care services to motivate pregnant women to utilise maternity services effectively and efficiently for the best pregnancy outcome.
